# Dual-Passband SAW Filter Based on a 32°YX-LN/SiO_2_/SiC Multilayered Substrate

**DOI:** 10.3390/mi14020479

**Published:** 2023-02-18

**Authors:** Huiping Xu, Sulei Fu, Rongxuan Su, Peisen Liu, Rui Wang, Fei Zeng, Cheng Song, Weibiao Wang, Feng Pan

**Affiliations:** 1 Key Laboratory of Advanced Materials (MOE), School of Materials Science and Engineering, Tsinghua University, Beijing 100084, China; 2 SHOULDER Electronics Limited, Wuxi 214214, China

**Keywords:** dual-band filter, multilayered substrate, surface acoustic wave, wireless communication

## Abstract

To meet the demands of highly integrated and miniaturized radio frequency front-end (RFFE) modules, multi-passband filters which support multi-channel compounding come to the foreground. In this work, we proposed a new design of a dual-passband surface acoustic wave (SAW) filter based on a 32°YX-LiNbO_3_ (LN)/SiO_2_/SiC multilayered structure. The filter is of a standalone ladder topology and comprises dual-mode resonators, in which the shear horizontal (SH) mode and high-order SH mode are simultaneously excited through electrode thickness modulation. The impact of electrode thickness on the performance of the dual-mode resonator was systematically investigated by the finite element method (FEM), and resonators were prepared and verified the simulation results. The electromechanical coupling coefficients (*K*^2^) of the SH modes are 15.1% and 17.0%, while the maximum *Bode-Q* (*Q*_max_) values are 150 and 247, respectively, for the fabricated resonators with wavelengths of 1 μm and 1.1 μm. In terms of the high-order SH modes in these resonators, the *K*^2^ values are 9.8% and 8.4%, and *Q*_max_ values are 190 and 262, respectively. The fabricated dual-band filter shows the center frequencies (*f*_c_) of 3065 MHz and 4808 MHz as two bands, with 3-dB fractional bandwidths (FBW) of 5.1% and 5.9%, respectively. Such a dual-band SAW filter based on a conventional ladder topology is meaningful in terms of its compact layout and diminished area occupancy. This work provides a promising avenue to constitute a high-performance dual-passband SAW filter for sub-6 GHz RF application.

## 1. Introduction

As 5G wireless communication depicts the blueprint of a smart life and the Internet of Everything (IoE), it also brings many challenges to the current radio frequency (RF) technology [1,2,3,4]. With the increasing number of frequency bands employed for long-term evolution (LTE) and global compatibilities, RF devices are required to be highly integrated and miniaturized as the volume of mobile handset is restricted [5,6,7]. As the essential component of RF front-end (RFFE) modules, filters stand at the forefront of innovation. Since conventional filters generally support a single passband, multi-passband filters which support multi-channel compounding are promising to reduce filter usage and thus realize the miniaturization of RFFE modules [8].

Many efforts have been made into researching multi-passband filters in recent years, especially the dual-band filter. The recently developed filter synthesis methodology provides the tools to better understand the interaction between the technological constraints and filter performance [9]. Taking advantage of the nodal approach, three methods to design dual-band filters with acoustic wave resonators are systematically discussed, and it was found that the conventional ladder topology can accurately control the final response and is compatible with the acoustic wave technology requirements [10]. K. V. Phani Kumar et al. presented a compact dual-band filter based on a signal interference technique, formed by the transmission lines [11]. They also achieved a dual-band filter based on a paper substrate, which was designed with a transversal filtering section, coupled lines, and stepped impedance stubs, showing high filtering selectivity and high isolation levels [12]. Zou et al. [13] demonstrated a dual-passband filter based on a film acoustic bulk resonator (FBAR) via simulation. The FBAR is composed of an AlN film with a *c*-axis-tilted angle, which leads to the coexistence of a longitudinal mode and a shear mode. Luo et al. [14] and Yang et al. [15] both proposed dual-band filters based on the modified lamb wave resonator (LWR), as the former one utilized two interdigital electrode (IDT) arrays with different thicknesses, and the latter one exploited the LN thickness difference. Dual-band surface acoustic wave (SAW) filters have been researched as well. By far, most of them tend to be a parallel connection of two independent filters, and thus the practical footprint is not reduced [16,17]. Among the abovementioned filter types, SAW exhibits better reproducibility, lower cost and a simpler fabrication process for mass production [18,19,20]. Therefore, the dual-passband SAW filter of a standalone ladder topology is of great significance to facilitate the integration and miniaturization of RFFE modules with low cost.

In this work, we proposed a novel dual-band SAW filter solution using a 32°YX-LiNbO_3_ (LN)/SiO_2_/SiC multilayered substrate. As the conventional SAW filter consists of merely IDTs and a LN/LiTaO_3_ (LT) bulk substrate, the emergent LN/LT thin film multilayered substrate provides multi-dimensional modulation possibilities for SAW designing [21,22,23]. Recently, wideband SAW filters based on Y-cut LN thin films were widely explored, which utilized the shear horizontal (SH) mode with large electromechanical coupling coefficient (*K*^2^) [24,25,26]. Furthermore, the high-order SH mode is found when the IDT electrode is thick, but its acoustic properties have not been studied in detail [27]. Here, the dual-mode resonator based on the simultaneous excitation of the SH mode and the high-order SH mode, through electrode thickness (*h*_e_) modulation, is successfully achieved. Accordingly, a dual-band SAW filter was fabricated showing center frequencies (*f*_c_) of 3065 MHz and 4804 MHz, respectively. Such a dual-band filter on a basis of a conventional ladder structure exhibits a compact layout and a small size in contrast to the dual-band filter using the double-ladder approach, making it a strong candidate in the dual-band filtering scheme [10,28]. This work provides a promising approach to constitute a dual-passband SAW filter for 5G application.

This article is organized as follows. Section 2 presents the theoretical investigation of the SH mode and the high-order SH mode of SAW resonators based on the above-proposed multilayered structure. Section 3 analyzes the properties of substrate materials and experimental results of the SAW devices fabricated according to the aforementioned simulations. Section 4 presents the conclusion.

## 2. Theoretical Investigation

**Figure 1:**
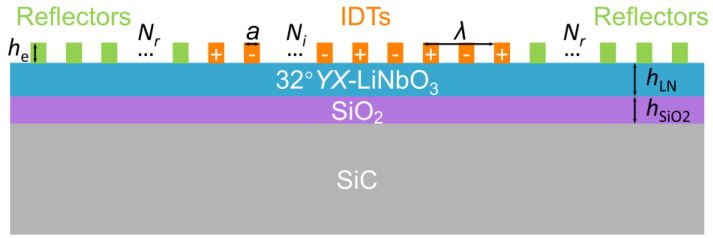
The main-view schematic of the one-port resonator.

Figure 1 shows the cross-sectional schematic of the resonator based on a 32°YX-LN/SiO_2_/SiC multilayered substrate. To explore the acoustic characteristics of the SH mode and the high-order SH mode, a 3D model of a unit block was established in the commercial software COMSOL Multiphysics 6.0, and finite element method (FEM) simulations were then carried out. In the simulation process, the wavelength (*λ*) is set to be 1 μm and the electrode material is aluminum (Al). The thickness of LN (*h*_LN_) and SiO_2_ (*h*_SiO2_) is equal to 0.3*λ* and 0.2*λ*, respectively. The selected *h*_LN_ and *h*_SiO2_ are beneficial for generating the SH mode and the high-order SH mode with higher acoustic velocity and *K*^2^.

**Figure 2:**
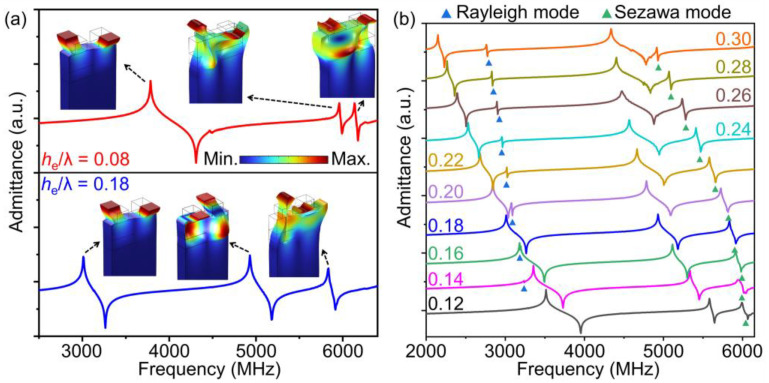
(**a**) The simulated admittance curves of the resonators with *h*_e_/*λ* = 0.08 and 0.18, respectively. The corresponding displacement schematic of each resonance is also calculated. (**b**) The simulated admittance curves with different *h*_e_/*λ*.

Figure 2a depicts the typical admittance curves with electrode-normalized thickness (*h*_e_/*λ*) = 0.08 and 0.18 and shows the displacement schematic of each resonance. Three resonances appear when *h*_e_/*λ* = 0.08. The SH mode is located at around 4 GHz, while two contiguous acoustic modes are observed at about 6 GHz. From the displacement schematics, the two contiguous modes exhibit the coupling characteristics of the high-order SH mode and the Sezawa mode. The two modes strongly interfere with each other, and their responses are both weak. Thus no high-frequency mode is capable of constituting the filter in this condition. As *h*_e_/*λ* increases to 0.18, the high-order SH mode and the Sezawa mode could be discriminated clearly, and the frequency interval between them is much larger. The high-order SH mode is able to be used in the filter at this time. Figure 2b shows a more detailed change of the admittance curve with the variation of *h*_e_/*λ*, and the spurious resonances (Rayleigh mode and Sezawa mode) are marked by the blue and green triangle symbols, respectively. It is observed that the SH mode is maintained regardless of electrode thickness, but the interference of the Rayleigh mode is worth noting. The Rayleigh mode will deteriorate the flatness of the passband formed by the SH mode, unless it could be eliminated or excluded far out of the band [29]. The response of the high-order SH mode is remarkably improved with increasing electrode thickness. However, the acoustic response of the high-order SH mode will be deteriorated as the *h*_e_/*λ* is larger than 0.28, which may result from an electrode that is too thick, leading to more energy dissipation in the electrode deformation. Meanwhile, the frequency interval between the high-order SH mode and the Sezawa mode increases at first and then decreases. The Sezawa mode will have little impact on the high-order SH mode within the researched *h*_e_/*λ*, as shown in Figure 2b, since the frequency interval between them is large enough.

**Figure 3:**
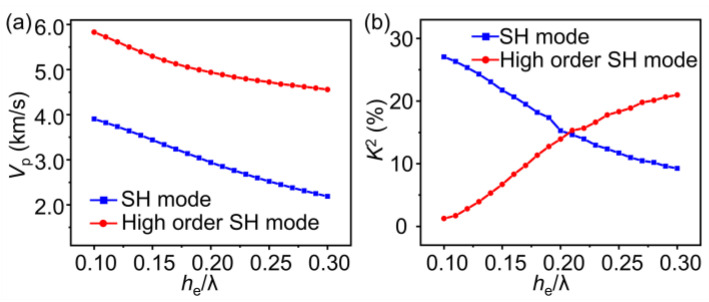
The simulated (**a**) *V*_p_ and (**b**) *K*^2^ of the SH mode and high-order SH mode as a function of *h*_e_/*λ*.

Figure 3a describes the phase velocity (*V*_p_) of the SH mode and high-order SH mode as a function of *h*_e_/*λ*. Here, *V*_p_ is estimated by *V*_p_ = (*f*_r_ + *f*_a_)*λ*/2, where *f*_r_ and *f*_a_ represent the resonance and anti-resonance frequencies, respectively [30]. The *V*_p_ of both modes decrease when *h*_e_/*λ* increases, and the SH mode shows a sharper downward trend than the high-order SH mode. When *h*_e_/*λ* increases from 0.18 to 0.28, the *V*_p_ declining rates of the SH mode and high-order SH mode are 35.6% and 8.6%, respectively. As the independent control of the resonance frequencies of two main modes is important for current adaptive spectrum allocation, such a discrimination in the *V*_p_ declining rate is beneficial for adjusting the frequency difference between the two main modes through modulating electrode thickness and wavelength.

*K*^2^ of both modes is also explored as shown in Figure 3b. The *K*^2^ of the high-order SH mode increases and the *K*^2^ of the SH mode decreases with *h*_e_/*λ* climbing. *K*^2^ is determined by the standard *IEEE* definition: *K*^2^ = (π*f*_r_/2*f*_a_)/tan(π*f*_r_/2*f*_a_) [31]. Therefore, a moderate *h*_e_/*λ* should be chosen when making a comprehensive consideration of the following three aspects: (1) enhancing the response quality of the high-order SH mode; (2) avoiding adverse effects of spurious modes; (3) maintaining a relatively large *K*^2^ value for both modes.

## 3. Device Measurement and Analysis

**Figure 4:**
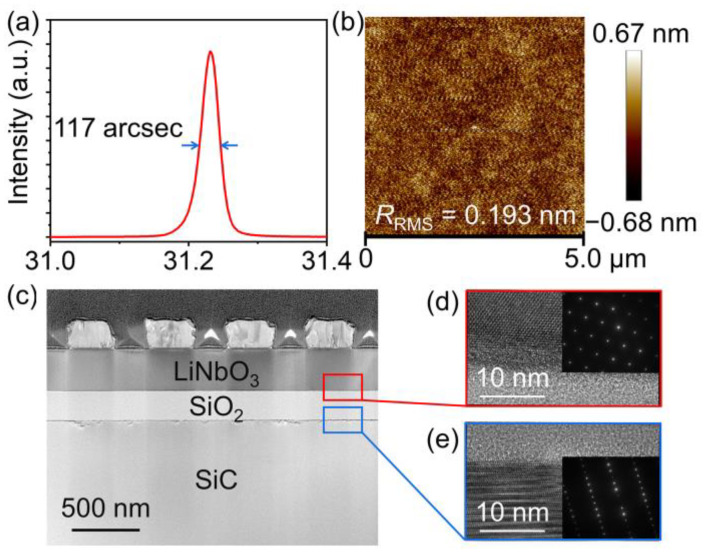
(**a**) XRD rocking curve of the (300) plane and (**b**) the AFM image of the 32°YX-LN film. (**c**) Cross-sectional TEM image of the resonator. HRTEM images of the interface of LN/SiO_2_ (**d**) and SiO_2_/SiC (**e**), and the insets are the SAED images of the LN and SiC layers.

After the optimization of the design parameters, the final multilayered structure is Al (195 nm)/Ti (5 nm)/32°YX-LN (300 nm)/SiO_2_ (200 nm)/4H-SiC, and Al (195 nm)/Ti (5 nm) serves as the IDT electrode layer. The selected electrode thickness balances, to an extent, the pursuit of high frequency and *K*^2^ with the processing difficulty. In terms of the preparation procedures, the SiO_2_ layer was first deposited on the highly insulated 4H-SiC substrate by magnetron sputtering. Helium ions (He^+^) were implanted into a 4-inch 32° Y-X LN wafer, thus forming a layer with defects below the surface of the LN wafer. Subsequently, the implanted wafer was bonded with the SiO_2_/4H-SiC substrate [32,33,34]. The 4-inch 32° Y-X LN wafer and 4-inch highly insulated 4H-SiC wafer are both commercial available. After bonding, the LN film was exfoliated along the defective layer via an annealing process. As the exfoliated surface was rough, the LN film was then trimmed and its thickness was reduced to 300 nm using the chemical–mechanical polishing technique. After fabricating the heterostructure, the thickness mapping images of the LN and SiO_2_ layer were tested using the spectrometer, showing thickness uniformities of both layers of around 5%. Finally, the IDT pattern was formed by the photolithography and lift-off process, and the electrode layer was deposited via e-beam evaporation. The crystal quality of the transferred LN layer strongly influences the SAW responses. Figure 4a depicts the rocking curve of the (300) plane (*y* plane) tested by high-resolution X-ray diffraction (XRD). The full width at half maximum (FWHM) is 117 arcsec. The root-mean-square surface roughness (*R*_RMS_) of the LN film is 0.193 nm, as shown in Figure 4b, characterized by the atomic force microscope (AFM). The small FWHM verifies the high crystal quality of the LN film, and the low *R*_RMS_ value indicates that the LN surface is smooth enough for SAW device fabrication [35]. Figure 4c shows the cross-sectional transmission electron microscope (TEM) image of the resonator. The TEM image demonstrates a straight and clear interface of LN-SiO_2_ and SiO_2_-SiC, and the measured *h*_e_, *h*_LN_ and *h*_SiO2_ are consistent with the expected values. The selected area electron diffraction (SAED) images and the clear lattice fringes from the high-resolution transmission electron microscope (HRTEM) (JEOL, Tokyo, Japan) images of the LN and SiC layers are described in Figure 4d,e, respectively, which further confirm the high-quality single-crystal characteristics of the LN and SiC layers.

**Figure 5:**
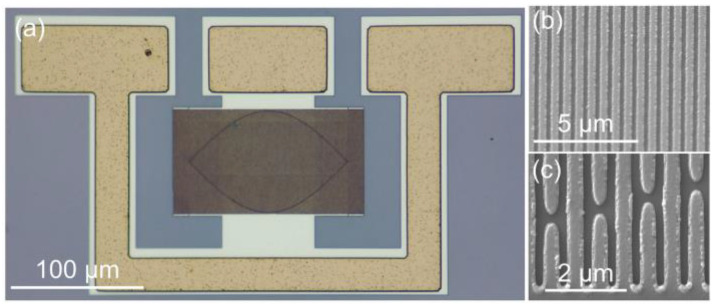
(**a**) Optical and (**b**,**c**) SEM images of the resonator with *λ* = 1.2 μm.

**Figure 6:**
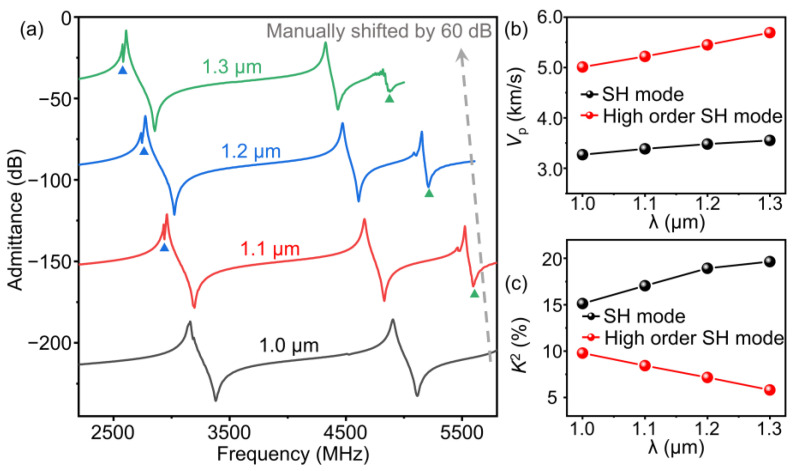
(**a**) The measured admittance curves of the dual-mode resonators with different *λ*. The Rayleigh mode and Sezawa mode are marked by the blue and green triangles, respectively. The measured *V*_p_ (**b**) and *K*^2^ (**c**) values of the SH mode and high-order SH mode at different *λ*.

Resonators with *λ* from 1 μm to 1.3 μm were fabricated on the 32°YX-LN/SiO_2_/SiC substrate. Figure 5a shows an optical microscope image of the resonator with *λ* = 1.2 μm. Here, the number of IDT fingers (*N*_i_) and reflector fingers (*N*_R_) is 200 and 20, respectively, and the IDT aperture is set to be 40*λ*. In order to suppress the transverse modes, a cosine-weighted IDT is utilized [36]. Figure 5b,c depict the scanning electron microscope (SEM) images of the IDT fingers from the above resonator with different magnifications. The electrodes show intact shapes and are regularly arranged. The measured admittance curves of the resonators are given in Figure 6a. Figure 6b,c summarize the measured *V*_p_ and *K*^2^ values of the SH mode and high-order SH mode at different *λ*. With the increase in *λ*, the *V*_p_ of the SH mode and high-order SH mode both climb. The *V*_p_ of the SH mode varies from 3269 to 3553 m/s, and the *V*_p_ of the high-order SH mode varies from 5010 to 5692 m/s. Meanwhile, the *K*^2^ value of the SH mode decreases and *K*^2^ value of the high-order SH mode increases when *h*_e_/*λ* ascends, and these trends are also consistent with the simulation results.

**Figure 7:**
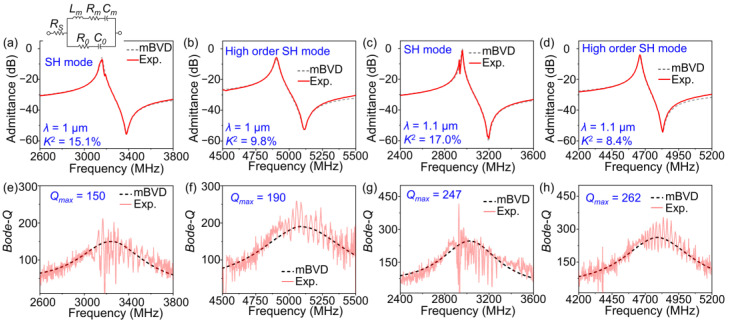
The measured and fitted admittance curves of the SH mode (**a**) and the high-order SH mode (**b**) of the resonator with *λ =* 1 μm. The measured and fitted *Bode-Q* curves of the SH mode (**c**) and the high-order SH mode (**d**) of the resonator with *λ =* 1 μm. The measured and fitted admittance curves of the SH mode (**e**) and the high-order SH mode (**f**) of the resonator with *λ =* 1.1 μm. The measured and fitted *Bode-Q* curves of the SH mode (**g**) and the high-order SH mode (**h**) of the resonator with *λ =* 1.1 μm.

To accurately extract the quality factors (*Bode*-*Q*) of the resonators, the modified Butterworth–Van Dyke (mBVD) equivalent circuit was introduced to fit the experimental results [37]. The mBVD model is shown in the inset of Figure 7a. Figure 7a–d depict the measured and mBVD-fitted admittance curves of the SH mode and the high-order SH mode with *λ* = 1 μm and 1.1 μm, respectively. The fitted curves overlap well with the measured curves, which confirms the reliability of the fitting results [38]. Figure 7e–h show the measured and fitted *Bode-Q* curves of the two modes with *λ* = 1 μm and 1.1 μm, respectively. When *λ* = 1 μm, the maximum *Bode-Q* (*Q*_max_) of the SH mode and the high-order SH mode are 150 and 190, respectively. As *λ* increases to 1.1 μm, *Q*_max_ of the SH mode and the high-order SH mode reach 247 and 262, respectively.

**Figure 8:**
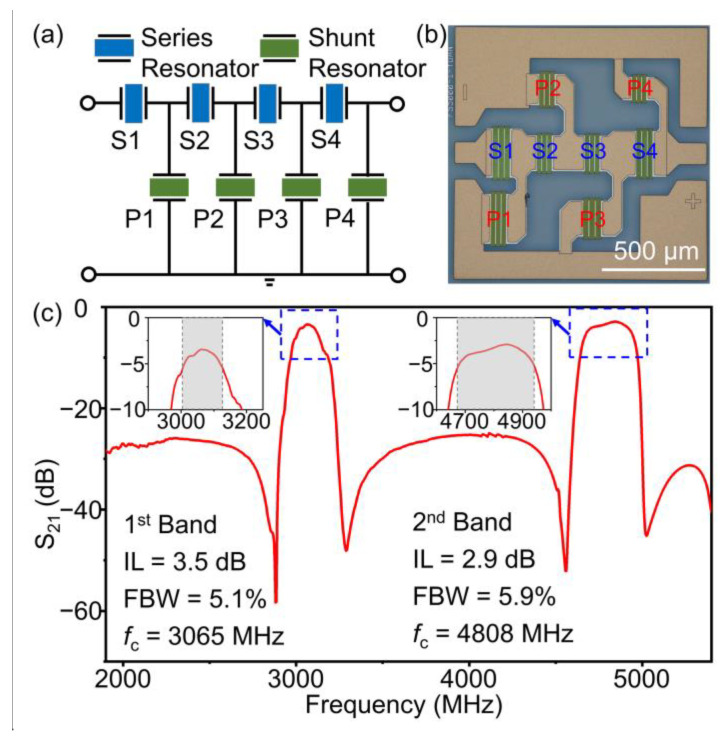
(**a**) The topology of the ladder-type SAW filter. (**b**) The optical microscope of the filter. (**c**) The measured S_21_ curve of the dual-band filter with *h*_e_ = 200 nm. The insets show magnified pictures of the S_21_ responses of the two bands, respectively, and the grey regions represent the 3-dB bandwidth.

Finally, a ladder-type dual-band filter was designed and fabricated. The topology of the filter is given in Figure 8a, and the corresponding optical microscope is depicted in Figure 8b. The structural parameters of the SAW filter are given in Table 1. Here, wavelengths of series resonators are adopted as 1 μm, while the wavelengths of the shunt resonators vary from 1.008 to 1.112 μm. Figure 8c shows the S_21_ curve of the filter. The first passband employing the SH mode shows an *f*_c_ of 3065 MHz, an insertion loss (IL) of 3.5 dB, and a 3-dB fractional bandwidth (FBW) of 5.1%. The second passband exploiting the high-order SH mode shows an *f*_c_ of 4808 MHz, an IL of 2.9 dB, and a 3-dB FBW of 5.9%. The band rejection is about 20 dB for both bands.

Here, the second band presents a better performance in terms of the bandwidth and loss than the first band, although the high-order SH mode has smaller *K*^2^ values and a higher frequency than the SH mode. The better performance of the second band should be attributed to the better quality factor of the high-order SH mode, which illustrates that the acoustic energy is leveraged more efficiently.

As this work aims at providing a feasible prototype for constructing a dual-band SAW filter, the adaptability for the detailed operational band is focused on less for the time being. For the further optimization to adapt to the practical spectrum, the designing parameters of the filter, such as thickness of the LN layer, electrode thickness, and wavelength, could be adjusted. For instance, when the *h*_e_/*λ* of the resonator reaches about 0.24, the resonant frequencies of the SH mode and the high-order SH mode are 2530 MHz and 4565 MHz, respectively, as shown in Figure 2b, which will meet the N41 and N79 bands under the circumstances. Hence, the appropriate dual-band filter in practical applications is promising in achieving the filtering function to two bands on its own, and further diminish the size of RFFE modules.

The importance of multi-channel compounding is increasing, thus dual-band filters will be highly needed in future communication systems. To this end, we have investigated and successfully achieved a dual-band filter on a basis of SAW resonators using a conventional ladder topology, which shows simple a fabrication procedure, diminished area occupancy, and compatibilities with acoustic wave technology requirements. Moreover, the center frequencies of two bands are beyond 3 GHz, meeting the frequency range of emerging sub-6 GHz applications.

## 4. Conclusions

This work proposed a novel design of a dual-passband SAW filter based on a 32°YX-LN/SiO_2_/SiC multilayered substrate. The filter is of a standalone ladder topology and comprises dual-mode resonators, in which the SH mode and the high-order SH mode were excited simultaneously through electrode thickness engineering. The impact of *h*_e_/*λ* on the performance of the dual-mode resonator was systematically investigated by FEM, and resonators with different *λ* were fabricated and verified the simulation results. The *K*^2^ values of the SH mode are 15.1% and 17.0%, while the *Q*_max_ values are 150 and 247, respectively, for fabricated resonators with wavelengths of 1 μm and 1.1 μm. In terms of the high-order SH modes in these resonators, the *K*^2^ values are 9.8% and 8.4%, and the *Q*_max_ values are 190 and 262, respectively. The fabricated SAW filter has two passbands located at 3065 MHz and 4808 MHz with 3-dB FBWs of 5.1% and 5.9%, respectively. The fine performance of the SAW devices of this work illustrates the feasibility of engaging the 32°YX-LN/SiO_2_/SiC structure into fabricating miniaturized dual-band filters for 5G application.

## Figures and Tables

**Table 1:** Structural parameters of SAW filter.

	S1	S2	S3	S4	P1	P2	P3	P4
*λ* (μm)	1.000	1.000	1.000	1.000	1.008	1.112	1.100	1.080
Aperture (μm)	21.90	18.57	18.57	20.59	19.14	19.06	22.93	17.55
*N*_i_	450	330	330	440	420	360	300	200
*N*_R_	20	20	20	20	20	20	20	20
*h*_e_/*λ*	0.200	0.200	0.200	0.200	0.198	0.180	0.182	0.185

## Data Availability

The data presented in this study are available on request from the corresponding author.
